# Standardized conversion model for retinal thickness measurements between spectral-domain and swept-source optical coherence tomography based on machine learning

**DOI:** 10.3389/fcell.2025.1612455

**Published:** 2025-07-17

**Authors:** Zhongping Tian, Yinning Guo, Xi Chen, Qifeng Zhou, Yuan Liu, Zhizhu Yi, Li Zhang

**Affiliations:** ^1^ Department of Ophthalmology, Tongji Hospital, School of Medicine, Tongji University, Shanghai, China; ^2^ School of Medicine, Tongji University, Shanghai, China; ^3^ Clinical Research Center, Tongji Hospital, School of Medicine, Tongji University, Shanghai, China; ^4^ School of Public Health, Zunyi Medical University, Zunyi, China

**Keywords:** optical coherence tomography, machine learning, retinal thickness, standardized conversion, spectral-domain, swept-source

## Abstract

**Purpose:**

To conduct a systematic comparative analysis of macular retinal thickness, retinal nerve fiber layer (RNFL) thickness, and ganglion cell-inner plexiform layer (GCIPL) thickness measurements between spectral-domain optical coherence tomography (SD-OCT) and swept-source OCT (SS-OCT) in healthy individuals, while establishing standardized cross-platform conversion algorithms through machine learning methodologies.

**Methods:**

In this cross-sectional investigation, 48 healthy adults (96 eyes) underwent macular retinal thickness assessment (ETDRS grid sectors), RNFL analysis (quadrant sectors), and GCIPL evaluation (six-sector annular divisions) using both SD-OCT (Cirrus HD-OCT 5000) and SS-OCT (Triton DRI-OCT). Inter-device measurement differences were evaluated through paired t-tests. Agreement metrics were quantified via intraclass correlation coefficients (ICCs) and Bland-Altman analysis. Four predictive models—linear regression (LR), LASSO regression, random forest regression (RF), and support vector regression (SVR)—were developed to estimate Triton DRI-OCT measurements from Cirrus HD-OCT 5000 outputs. Model efficacy was assessed using coefficient of determination (R^2^) and root mean square error (RMSE).

**Results:**

Statistically significant inter-device discrepancies (P < 0.001) were identified in 9 macular sectors, all GCIPL parameters (average and six-sector measurements), and RNFL measurements (average thickness and three quadrants, excluding nasal sector). ICC values demonstrated moderate-to-strong agreement: macular thickness (0.771–0.906), GCIPL (0.554–0.710), and RNFL (0.451–0.852). Machine learning models exhibited superior performance in central subfield thickness (CST) prediction, achieving test set R^2^ values of 0.930 (LR), 0.926 (LASSO), 0.936 (SVR), and 0.892 (RF). Linear regression maintained consistent predictive accuracy across parameters: CST (R^2^ = 0.930), RNFL (R^2^ = 0.845), and GCIPL (R^2^ = 0.760).

**Conclusion:**

Substantial measurement discrepancies preclude direct interchangeability of SD-OCT and SS-OCT datasets. Machine learning-derived conversion algorithms significantly improve cross-device comparability, offering a robust standardization framework for multicenter research and longitudinal data integration. This methodological advancement enables harmonized analysis of OCT metrics across heterogeneous imaging platforms.

## 1 Introduction

Optical coherence tomography (OCT) is a non-invasive imaging technology widely used in ophthalmic diagnostics, particularly for retinal and optic nerve evaluation (Huang et al., 1991). Compared to traditional imaging, OCT offers superior resolution and speed, enabling real-time visualization of microstructural changes for early disease detection (Costa et al., 2006). Beyond ophthalmology, OCT has shown potential in systemic diseases such as Alzheimer’s and Parkinson’s (Gaire et al., 2024; Ge et al., 2021; Wagner et al., 2023).

Advancements in OCT technology have led to spectral-domain OCT (SD-OCT) and swept-source OCT (SS-OCT), which differ in scanning speed, penetration depth, and segmentation algorithms (Laíns et al., 2021). These differences result in significant inter-device variations in key metrics like central subfield thickness (CST) (Nam et al., 2024; Matlach et al., 2014), retinal nerve fiber layer (RNFL) (Matlach et al., 2014; Pierro et al., 2012; Seibold et al., 2010), ganglion cell inner plexiform layer (GCIPL) (Mahmoudinezhad et al., 2021), limiting cross-study data integration (Tan et al., 2016). Standardized conversion equations are thus critical for harmonizing multi-device datasets (Bressler et al., 2014; Lee et al., 2021; Schrems et al., 2015; Shepherd et al., 2012).

Artificial intelligence (AI) has emerged as a powerful tool in ophthalmology (Foo et al., 2021; Wang et al., 2023; Shahriari et al., 2023; Feng et al., 2023; Li et al., 2024; Xu et al., 2022; Xu et al., 2020). Methods based on machine learning or deep learning can learn patterns or regularities from data through algorithms, achieving robust predictive or decision-making performance. At present, Cirrus HD-OCT 5000 and Triton DRI-OCT are the SD-OCT and SS-OCT, respectively, commonly used in clinical practice, and the studies on the measurement differences between the two machines are mostly limited to a certain amount of measurement indexes and do not give a clear method of data standardization (Nam et al., 2024; Lee et al., 2017). Therefore, this study is the first to jointly evaluate the differences between SD-OCT and SS-OCT for whole-zone measurements of macular retinal thickness, RNFL, and GCIPL, and to construct machine learning-based standardized equations to address data compatibility issues in clinical studies and multicenter collaborations.

## 2 Materials and methods

### 2.1 Study population

Healthy adults (n = 48, 96 eyes) were recruited from Tongji Hospital, Shanghai, between October and November 2024. All participants underwent at least one Cirrus HD-OCT 5000 and Triton DRI-OCT examination at the Department of Ophthalmology. Exclusion criteria included ocular pathology (except refractive errors: myopia <−6D, hyperopia <+3D), congenital anomalies, prior ocular surgery, or poor scan quality (Cirrus signal strength <5/10; Triton <45/100).

### 2.2 Ocular examinations

The Cirrus HD-OCT 5000 (Carl Zeiss MediaTek, Dublin, CA) was used to obtain a volumetric scan of the macula containing a 6 mm × 6 mm area of the macular center pits by means of a Macular Cube 512 × 128 (6 mm × 6 mm) scan, and the retinal thickness of the macula was defined as the vertical distance from the inner limiting membrane (ILM) to the retinal The vertical distance from the ILM to the middle layer of the retinal pigment epithelium (RPE), CST was expressed as the average thickness of the retina with a central diameter of 1 mm in the macula, macular GCIPL thickness was measured as the sum of the thicknesses of the ganglion cell layer and the inner tufted layer in a 4 mm × 5 mm ring surrounding the macular central concavity, and the Optic Disc Cube 200 × 200 scan was used to measure RNFL thicknesses in the circular region centered on the optic disc with a diameter of 3.46 mm.

Triton DRI-OCT (Topcon, Inc, Tokyo, Japan) was used to obtain a macular volumetric scan containing an area >6 mm around the macular central concavity by means of a Macular Cube 512 × 128 (6 mm × 6 mm) scan, with the retinal thickness of the macula defined as the perpendicular distance from the ILM to the boundary of the outer photoreceptor segment (OS) and the RPE (7), macular GCIPL thickness was measured as the sum of the thickness of the ganglion cell layer and the inner plexiform layer in a 6 mm × 6 mm cylindrical region containing the 6 mm × 6 mm area surrounding the macular central concavity, and an Optic Disc Cube 200 × 200 scan was used to measure the thickness of the RNFL in a circular region of 3.4 mm in diameter centered on the optic disk (Lee et al., 2017).

For macular retinal thickness, the area was divided using the Early Treatment Diabetic Retinopathy Study (ETDRS) grid (Jin et al., 2016) and the average regional thickness was calculated for each sector in the ETDRS grid. For macular GCIPL thickness, the measurement area was averaged into six sectors, with the Cirrus automatically measuring the average, minimum, and six sector GCIPL thicknesses and the Triton automatically measuring the average and six sector GCIPL thicknesses. For RNFL thickness, the measurement area was averaged into four quadrants, superior, inferior, temporal and nasal, with both devices automatically measuring the total area and the average RNFL thickness in each quadrant.

### 2.3 Quality control

All participants had two OCT scans completed at the same location on the same day. Patients with signal intensity Cirrus HD-OCT 5000 < 5/10, Triton DRI-OCT <45/100 or poor image quality were retaken. All OCT scans followed the OSCAR-IB standard for scan quality control (Aytulun et al., 2021; Cruz-Herranz et al., 2016). Segmentation of retinal layers was performed using inbuilt software, and OCT image segmentation levels were manually reviewed and modified by trained technicians and clinicians to ensure compliance with quality control standards.

### 2.4 Statistical analysis

Data were analyzed using SPSS (SPSS, Inc., Chicago, IL, version 20.0). Measurements were described as mean ± standard deviation or median and interquartile spacing, and comparisons between different machines were performed using paired-samples t-tests. Bland-Altman plots and intraclass correlation coefficients (ICC) were used to assess the consistency of measurements between machines. R statistical software (version 4.3.1; R Core Team, 2023) was used to construct the machine learning models. *P* < 0.05 was considered statistically significant.

Participants were divided into a training set and a testing set according to a ratio of 8:2. The training set data were used to construct the model and the testing set data were used to validate the reliability of the model. Outliers were excluded from the derivation of the conversion equations, which were defined as the difference in CST, RNFL, or GCIPL measurements between two devices that differed from the average difference by > 2 SD. Four machine learning methods, namely, linear regression (LR), LASSO regression, Random Forest regression (RF), and Support Vector Machine regression (SVR) were utilized to develop conversion equations for CST, RNFL, and GCIPL thicknesses, respectively, to convert the measured values of Cirrus HD-OCT 5000 were converted to predicted values of Triton DRI-OCT. The reliability of the model was evaluated by calculating the coefficient of determination (R^2^), root mean square error (RMSE).

## 3 Results

A total of 96 eyes of 48 healthy adults (including 13 males and 35 females) with a mean age of 32.9 ± 13.95 years were included in this study.

### 3.1 Inter-device differences

Among the quantitative fundus indices measured with both the Cirrus HD-OCT 5000 and Triton DRI-OCT machines, except for the nasal sector of the RNFL thickness, the measurements of each division of the macular retina, the GCIPL, and the remaining divisions of the RNFL were significantly different between the two groups (P < 0.001, Table 1). The CST (245.4 ± 19.79 μm vs. 231.2 ± 16.52 μm) and GCIPL (82.9 ± 6.58 μm vs. 64.1 ± 4.20 μm) of Cirrus HD-OCT 5000 were significantly higher than Triton DRI-OCT, whereas the mean RNFL thickness (97.8 ± 10.01 μm vs. 106.4 ± 10.51 μm) was significantly lower.

**TABLE 1:** Comparison of retinal thickness measurements between SD-OCT and SS-OCT.

Retinal measurements (μm)	HD-OCT 5000	DRI-OCT	*P*
Macular retinal thickness			
CST	245.4 ± 19.79	231.2 ± 16.52	<0.001^***^
Inner			
Superior	321.5 ± 16.97	307.9 ± 13.85	<0.001^***^
Temporal	306.1 ± 14.35	294.3 ± 13.21	<0.001^***^
Inferior	312.6 ± 15.18	303.1 ± 13.70	<0.001^***^
Nasal	320.2 ± 15.80	306.3 ± 14.25	<0.001^***^
Outer			
Superior	285.9 ± 13.89	272.0 ± 12.71	<0.001^***^
Temporal	263.6 ± 11.72	252.1 ± 11.95	<0.001^***^
Inferior	264.8 ± 13.73	254.9 ± 12.72	<0.001^***^
Nasal	300.9 ± 15.65	286.4 ± 14.53	<0.001^***^
GCIPL			
Mean thickness	82.9 ± 6.58	64.1 ± 4.20	<0.001^***^
Superior	83.8 ± 8.47	70.34 ± 4.95	<0.001^***^
Supertemporal	82.2 ± 7.05	70.5 ± 4.91	<0.001^***^
Inferotemporal	82.3 ± 7.39	71.0 ± 4.76	<0.001^***^
Inferior	78.7 ± 9.74	65.3 ± 4.78	<0.001^***^
Inferonasal	82.7 ± 8.22	71.8 ± 5.23	<0.001^***^
Super nasal	85.6 ± 8.14	74.4 ± 5.61	<0.001^***^
RNFL			
Mean thickness	97.8 ± 10.01	106.4 ± 10.51	<0.001^***^
Superior	114.0 ± 18.52	125.7 ± 18.93	<0.001^***^
Temporal	88.5 ± 18.44	101.4 ± 22.48	<0.001^***^
Inferior	125.4 ± 19.35	137.7 ± 22.70	<0.001^***^
Nasal	63.3 ± 13.91	61.0 ± 19.82	0.198

Note: CST, central subfield thickness; GCIPL, ganglion cell inner plexiform layer; RNFL, retinal nerve fiber layer; Data are presented as mean ± SD.

*P < 0.05, **P < 0.01, ***P < 0.001.

### 3.2 Inter-device agreement analysis

**FIGURE 1:**
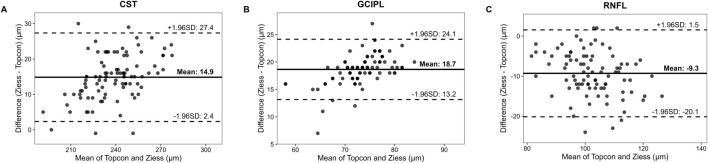
The Bland-Altman Plots Comparing Retinal Thickness Measurements between SD-OCT and SS-OCT. Notes: Solid line means the mean difference between Cirrus HD-OCT 5000 (SD-OCT) and Triton DRI-OCT (SS-OCT) measurements; Dashed lines means 95% limits of agreement (mean difference ±1.96 × standard deviation). Data points represent paired measurements from 96 eyes. CST **(A)** Mean difference = 14.9 ± 12.5 μm. A systematic bias is evident, with SD-OCT consistently measuring higher CST values than SS-OCT; GCIPL **(B)** Mean difference = 18.7 ± 5.5 μm. Narrower limits of agreement suggest better reproducibility for GCIPL compared to RNFL; RNFL **(C)** Mean difference = −9.3 ± 10.8 μm. Wider dispersion of points indicates poorer agreement, particularly in the nasal quadrant (ICC = 0.451).

The ICC distribution of macular retinal thickness between the two groups of Cirrus HD-OCT 5000 and Triton DRI-OCT ranged from 0.771 to 0.906 (*P* < 0.001), as shown in Table 2. The lowest ICC was found in the upper outer ring of the macular retinal thickness (0.771, *P* < 0.001), and the highest ICC was found in the lower outer ring and the nasal region (0.906, *P* < 0.001). The ICC indices of GCIPL thickness were distributed between the two groups between 0.554 and 0.710 (*P* < 0.001), with the ICC of the average GCIPL thickness being the highest. The ICC indices of RNFL thickness were distributed between the two groups between 0.451 and 0.852 (*P* < 0.001), with the thickness of the RNFL nasal sector having the ICC was the lowest (0.451, *P* < 0.001), as shown in Table 2. Bland-Altman analysis showed that the mean deviations between groups were 14.9 ± 12.5 μm, 18.7 ± 5.5 μm, and −9.3 ± 10.8 μm for the CST, GCIPL, and RNFL, respectively (Figure 1).

**TABLE 2:** The ICC of retinal thickness measurements between SD-OCT and SS-OCT.

Retinal measurements (μm)	HD-OCT 5000	DRI-OCT	ICC (95%CI)	*P*
Macular retinal thickness				
CST	245.4 ± 19.79	231.3 ± 16.52	0.890 (0.839–0.926)	<0.001^***^
Inner				
Superior	321.5 ± 16.97	307.9 ± 13.85	0.838 (0.765–0.889)	<0.001^***^
Temporal	306.1 ± 14.35	294.3 ± 13.21	0.878 (0.822–0.917)	<0.001^***^
Inferior	312.6 ± 15.18	303.1 ± 13.70	0.887 (0.835–0.924)	<0.001^***^
Nasal	320.2 ± 15.80	306.3 ± 14.25	0.853 (0.787–0.900)	<0.001^***^
Outer				
Superior	285.9 ± 13.89	272.0 ± 12.71	0.771 (0.674–0.842)	<0.001^***^
Temporal	263.6 ± 11.72	252.1 ± 11.95	0.865 (0.803–0.909)	<0.001^***^
Inferior	264.8 ± 13.73	254.9 ± 12.72	0.906 (0.861–0.936)	<0.001^***^
Nasal	300.9 ± 15.65	286.4 ± 14.53	0.906 (0.861–0.936)	<0.001^***^
GCIPL				
Mean thickness	82.9 ± 6.58	64.1 ± 4.20	0.710 (0.592–0.798)	<0.001^***^
Superior	83.8 ± 8.47	70.34 ± 4.95	0.604 (0.459–0.718)	<0.001^***^
Supertemporal	82.2 ± 7.05	70.5 ± 4.91	0.603 (0.458–0.717)	<0.001^***^
Inferotemporal	82.3 ± 7.39	71.0 ± 4.76	0.609 (0.465–0.721)	<0.001^***^
Inferior	78.7 ± 9.74	65.3 ± 4.78	0.554 (0.397–0.679)	<0.001^***^
Inferonasal	82.7 ± 8.22	71.8 ± 5.23	0.695 (0.574–0.786)	<0.001^***^
Super nasal	85.6 ± 8.14	74.4 ± 5.61	0.681 (0.557–0.776)	<0.001^***^
RNFL				
Mean thickness	97.8 ± 10.01	106.4 ± 10.51	0.743 (0.639–0.821)	<0.001^***^
Superior	114.0 ± 18.52	125.7 ± 18.93	0.757 (0.657–0.831)	<0.001^***^
Temporal	88.5 ± 18.44	101.4 ± 22.48	0.700 (0.582–0.790)	<0.001^***^
Inferior	125.4 ± 19.35	137.7 ± 22.70	0.852 (0.786–0.898)	<0.001^***^
Nasal	63.3 ± 13.91	61.0 ± 19.82	0.451 (0.277–0.597)	<0.001^***^

Notes: CST, central subfield thickness; RNFL, retinal nerve fiber layer; GCIPL, ganglion cell-inner plexiform layer; CI, confidence interval; SD-OCT, spectral-domain optical coherence tomography; SS-OCT, swept-source optical coherence tomography. Values are presented as mean ± standard deviation. ICC, interpretation; ICC, values were calculated using a two-way mixed-effects model for absolute agreement. *P* values indicate significance levels for ICC, comparisons between devices.

*P < 0.05, **P < 0.01, ***P < 0.001.

### 3.3 Model performance

**FIGURE 2:**
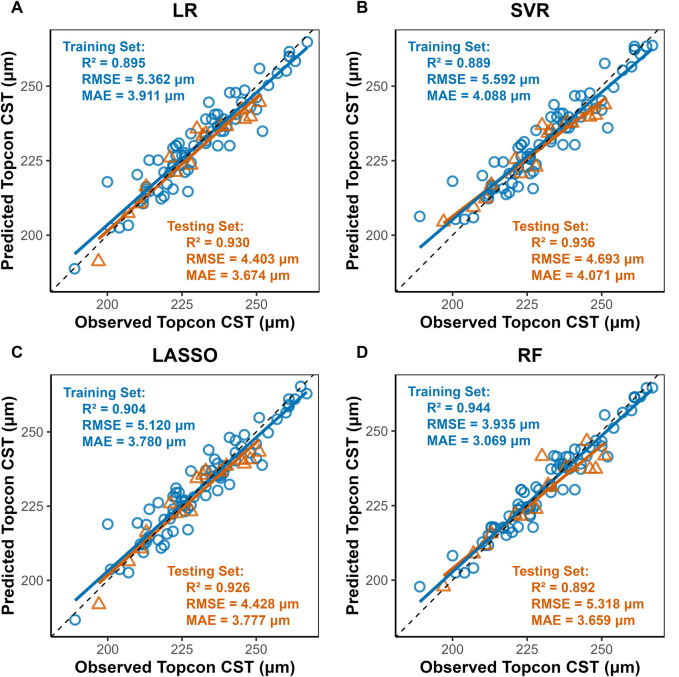
Scatter Plots of Observed vs. Predicted CST Values by Machine Learning Models. Notes: LR, Linear Regression; LASSO, Least Absolute Shrinkage and Selection Operator; SVR, Support Vector Regression; RF, Random Forest. X-axis, Predicted CST values (μm) from Triton DRI-OCT (SS-OCT); Y-axis: Observed CST values (μm) from Cirrus HD-OCT 5000 (SD-OCT). Diagonal line: Ideal agreement line (y = x), where predicted values perfectly match observed values. Data points: Each point represents an individual eye measurement (n = 96). **(A)** LR: Clustered tightly around the diagonal line, indicating high predictive accuracy (test R^2^ = 0.930). **(B)** SVR: Slightly wider dispersion in the test set (RMSE = 4.693) but retains strong correlation (R^2^ = 0.936). **(C)** LASSO: also indicating high predictive accuracy (test R^2^ = 0.930) **(D)** RF: Training set points (blue) align closely with the line (R^2^ = 0.944), while test set (yellow) shows moderate deviation (R^2^ = 0.892), suggesting mild overfitting.

**FIGURE 3:**
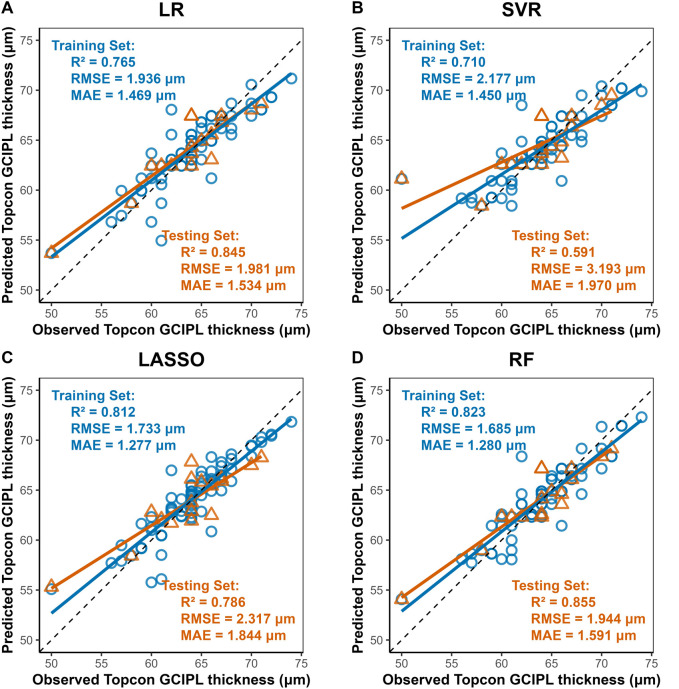
Scatter Plots of Observed vs. Predicted GCIPL Average Thickness by Machine Learning Models. Notes: X-axis, Predicted GCIPL thickness (μm) from Triton DRI-OCT (SS-OCT). Y-axis, Observed GCIPL thickness (μm) from Cirrus HD-OCT 5000 (SD-OCT). Diagonal line: Ideal agreement line (y = x), representing perfect prediction accuracy. Data points: Each point corresponds to an individual eye measurement (n = 96). **(A)** LR: LR shows moderate generalizability (test R^2^ = 0.845). **(B)** SVR: Exhibits the poorest performance (test R^2^ = 0.591, RMSE = 3.193 μm), with significant dispersion in both training and test sets. **(C)** LASSO: LASSO’s regularization slightly reduces overfitting compared to SVR **(D)** Random Forest (RF): Demonstrates the best overall performance with the highest test set R^2^ (0.855) and lowest test RMSE (1.944 μm), closely aligning with the diagonal line.

**FIGURE 4:**
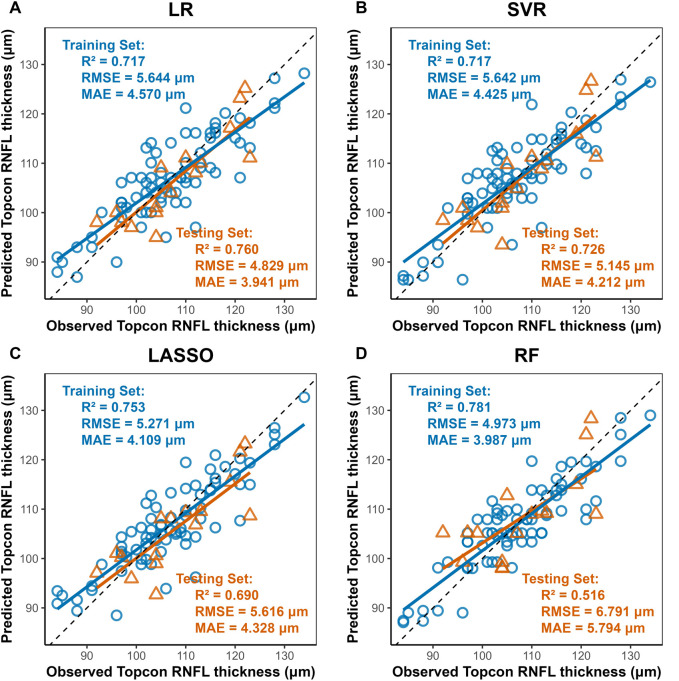
Scatter Plots of Observed vs. Predicted RNFL Average Thickness by Machine Learning Models. Notes: Axes: X-axis, Predicted RNFL thickness (μm) from Triton DRI-OCT (SS-OCT). Y-axis, Observed RNFL thickness (μm) from Cirrus HD-OCT 5000 (SD-OCT). Diagonal line: Ideal agreement line (y = x), indicating perfect prediction accuracy. Data points: Each point represents an individual eye measurement (n = 96). **(A)** Linear Regression (LR): Achieves the highest test set R^2^ (0.760) and lowest test RMSE (4.829 μm), with data points clustered near the diagonal line, demonstrating robust generalizability. **(B,C)** LASSO and SVR: Intermediate performance (test R^2^ = 0.690–0.726), with SVR slightly outperforming LASSO in test RMSE (5.145 μm vs. 5.616 μm). **(D)** Random Forest (RF): Shows the lowest test performance (R^2^ = 0.516, RMSE = 6.791 μm), with significant deviation in the test set (yellow), indicating pronounced overfitting despite high training accuracy (R^2^ = 0.781).

Four machine learning methods were used to construct prediction models about the thickness of CST, RNFL, and GCIPL, respectively, and the results showed that the performance of the four models was similar between training set and testing set, and the nonlinear model was not significantly better than the linear model, see Table 3. For CST, RF was the best in the training set (RMSE = 3.935, R^2^ = 0.944), and SVR was the best in the testing set (RMSE = 4.693, R^2^ = 0.936), see Figure 2, and for GCIPL, RF was the best in predicting GCIPL, with an RMSE of 1.685 in the training set, an R^2^ of 0.823, and in the testing set an RMSE of 1.944 and R^2^ of 0.855, see Figure 3; whereas the four models generally have low prediction performance on RNFL (R^2^ = 0.516–0.760), with RF performing the best in the training set (RMSE = 4.973, R^2^ = 0.781), and LR in the testing set (RMSE = 4.829, R^2^ = 0.760), the see Figure 4.

**TABLE 3:** Model performance on Various clinical Indicators.

Metrics	Model	Training set		Testing set	
		RMSE	R^2^	RMSE	R^2^
CST	LR	5.362	0.895	4.403	0.930
	LASSO	5.120	0.904	4.428	0.926
	SVR	5.592	0.889	4.693	0.936
	RF	3.935	0.944	5.318	0.892
GCIPL	LR	1.936	0.765	1.981	0.845
	LASSO	1.733	0.812	2.317	0.786
	SVR	2.177	0.710	3.193	0.591
	RF	1.685	0.823	1.944	0.855
RNFL	LR	5.644	0.717	4.829	0.760
	LASSO	5.271	0.753	5.616	0.690
	SVR	5.642	0.717	5.145	0.726
	RF	4.973	0.781	6.791	0.516

Notes: CST, central subfield thickness; GCIPL, Ganglion Cell-Inner Plexiform Layer; RNFL, Retinal Nerve Fiber Layer. LR, linear regression; LASSO, least absolute shrinkage and selection operator; SVR, support vector regression; RF, Random Forest. RMSE, root mean square error; R^2^, Coefficient of Determination. RMSE, Lower values indicate better predictive accuracy. R^2^, Higher values (closer to 1) indicate better model fit. Models were evaluated using an 80:20 split for training and test sets. Hyperparameters were optimized via cross-validation on the training set.

## 4 Discussion

In this study, we found that there were large differences in the measurements between Triton DRI-OCT and Cirrus HD-OCT 5000 between macular retinal thickness in the macula, macular GC-IPL thickness, and RNFL thickness, which is consistent with previous studies (Nam et al., 2024; Lee et al., 2017). There are multiple reasons for such measurement differences, including differences in SS-OCT and SD-OCT scanning parameters, image segmentation algorithms, and measurement areas (Laíns et al., 2021; Pierro et al., 2012; Heussen et al., 2012), such as in the measurement of the CST, the Cirrus HD-OCT 5000 when measuring the distance from the ILM to the intermediate layer of the RPE, and the Triton DRI-OCT when measuring the distance from the ILM to the OS and the RPE boundaries, and when measuring RNFL thickness, the Cirrus HD-OCT 5000 measured the average RNFL thickness in a 3.46 mm diameter circular area centered on the optic disk, while the Triton DRI-OCT measured a 3.4 mm diameter circular area (Lee et al., 2017).

We also compared the consistency of the measurements between the two devices, which did not have consistent ICC indices between each measurement, with the lowest ICC index located in the thickness of the nasal sector of the RNFL (0.451, P < 0.001) and the highest ICC index in the thickness of the outer subcircular region of the macular retina and the nasal region (0.906, *P* < 0.001). In contrast, Nam et al. (2024) found that the Triton OCT-1 and Cirrus HD-OCT 5000 had a higher ICC index in the temporal region of the outer retinal ring in the macula (0.930, 95% CI 0.828–0.972), which may be related to the population differences and segmentation algorithms between the machines. Overall, both devices showed high agreement in measured retinal thickness, RNFL thickness, and GCIPL thickness in the macular region.

Nonetheless, the significant differences between the two devices on the same measurements resulted in their measurements not being directly merged for use. Therefore, standardized conversion of data in clinical studies involving multiple OCT devices is mandatory (Shepherd et al., 2012; Kenney et al., 2022). Based on four machine learning methods, we constructed a model for standardized conversion of OCT measurement data, in which the model showed the best predictive performance for CST, with R^2^ ranging from 0.892 to 0.930 in the testing set, and the worst predictive performance for RNFL, with R^2^ ranging from 0.516 to 0.760 in the testing set, which may be mainly due to the difference between the two machines in the RNFL and the GCIPL with different segmentation criteria (Lee et al., 2017), which resulted in the measures showing large differences. Moreover, it is noteworthy that the study cohort exclusively comprised healthy adults, whose clear and regular retinal images do not currently challenge OCT segmentation algorithms. However, in patients with ocular pathologies such as diabetic macular edema (DME) or wet age-related macular degeneration (wAMD), irregular retinal structures frequently induce OCT segmentation errors. This may lead to significant discrepancies in measurements between different devices. Consequently, researchers have initiated efforts to incorporate diseased populations—specifically establishing data conversion equations for CST in DME patients (Bressler et al., 2014; Bressler et al., 2015; Ibrahim et al., 2012), and developing conversion equations for macular retinal thickness in AMD patients (Krebs et al., 2011). Therefore, some researchers have pinned their hopes on reducing the measurement differences of multiple OCT devices by constructing a standardized automatic segmentation model based on Cycle GAN for image segmentation and layering of multiple OCT devices (Chen et al., 2023), but this research is still affected by the inconsistent image resolution and scanning artifacts of different OCT devices. Building upon the above discussion, while the data conversion equations derived from healthy retinae in this study require further validation for extension to patients with fundus diseases, this conversion model holds substantial potential for research on retinal alterations associated with extraocular pathologies such as Alzheimer’s disease (AD), mild cognitive impairment (MCI), multiple sclerosis (MS), and cerebrovascular disorders.

Among these four machine learning models, this study observes that LR is more stable in its performance on CST, RNFL, and GCIPL in both training and testing sets, and the R^2^ in the testing set is 0.930, 0.845, and 0.760. This finding aligns with previous research outcomes. Schrems et al. (2015), established conversion equations for RNFL thickness between SD-OCT and TD-OCT using multiple methodologies, including non-conversion, mean difference, mean ratio, and linear regression approaches. Among these, linear regression demonstrated optimal predictive performance. Although numerous prior studies have endeavored to develop OCT data conversion equations(9, 29), few have employed machine learning paradigms such as nonlinear modeling. Our results similarly indicate that nonlinear models confer no significant advantage in constructing data conversion equations. Therefore, constructing the data conversion equation between two OCT machines using a linear model is a simple and effective standardization method. On the other hand, previous studies on OCT data conversion have been largely confined to comparisons between SD-OCT and TD-OCT. Kenney et al. (2022), through a generalized linear estimation model proposed data transformation equations for Cirrus and Spectralis SD-OCT on pRNFL and GCIPL, i.e., Cirrus = −5.0 + 1.0 × Spectralis pRNFL and Cirrus = −4.5 + 0.9 × Spectralis GCIPL; Bressler et al. (2014) constructed data conversion equations for the CST of patients with diabetic macular edema measured using TD-OCT and SD-OCT, which were found to further reduce measurement error. Sun et al. (2021), on the other hand, used a linear mixed model to construct the CST of patients with diabetic macular edema between the Cirrus and Spectralis SD-OCT transformation equation, i.e., Spectralis = 40.78 + 0.95 × Cirrus, and good predictive performance was also observed. This study concentrates on measurement discrepancies between SS-OCT and SD-OCT. Following the development of data conversion equations, empirical validation demonstrates their capacity to substantially mitigate these inter-device variations.

This study posits that data conversion equations can address two critical issues in clinical research. Firstly, the integration of OCT data in multicenter studies: Data conversion equations enable standardized transformation of raw measurements from different OCT devices, allowing merged analysis or comparison of standardized multicenter data to minimize measurement discrepancies. Secondly, OCT data standardization in longitudinal follow-up studies: Equipment replacement during extended follow-up introduces significant measurement errors that compromise data integrity; applying these conversion equations permits standardization of historical data in select studies, thereby mitigating inter-device variability. While maintaining consistent measurement protocols remains the gold standard for robust clinical research, these conversion equations must be judiciously applied when unavoidable technical transitions occur.

Nonetheless, the limitations of this study are as follows: only healthy adults were included in this study, and differences between race, age (Tham et al., 2020) and disease (Bressler et al., 2014) were not considered, and the applicability of the constructed data conversion model to patients with macular or optic nerve disease still requires further investigation; in addition, the conversion equations for data measured by different machines are different (Bressler et al., 2014; Lee et al., 2021; Schrems et al., 2015), and the data conversion model constructed in this study is limited to the Triton DRI-OCT and Cirrus HD-OCT 5000 machines; furthermore, the data conversion model constructed to combine the measurement data from different OCT devices still has overfitting or underfitting, which requires caution in actual clinical research, but there are certain differences in scanning parameters, image segmentation algorithms, and measurement areas between the devices. However, there are some differences in scanning parameters, image segmentation algorithms and measurement areas between devices, and it is still helpful to construct a data conversion model to minimize the measurement error in clinical research.

## 5 Conclusion

Our study found that there are significant differences in macular retinal thickness, GCIPL, and RNFL thickness between the Triton DRI-OCT and the Cirrus HD-OCT 5000, and the construction of a machine learning model can effectively reduce this measurement error, which provides a reliable data standardization method for cross-sectional studies or long-term follow-up studies using different OCT devices.

## Data Availability

The data analyzed in this study is subject to the following licenses/restrictions: Healthy People Data Measured Using Both Cirrus HD-OCT 5000 and Triton DRI-OCT Devices. Requests to access these datasets should be directed to Zhongping Tian, tianzp0814@163.com.
